# Premature Mortality in Type 2 Diabetes Mellitus Associated with Heart Failure and Chronic Kidney Disease: 20 Years of Real-World Data

**DOI:** 10.3390/jcm11082131

**Published:** 2022-04-11

**Authors:** Cristina Gavina, Daniel Seabra Carvalho, Daniel Martinho Dias, Filipa Bernardo, Hugo Martinho, João Couceiro, Carla Santos-Araújo, Ricardo Jorge Dinis-Oliveira, Tiago Taveira-Gomes

**Affiliations:** 1 Cardiology Department, Pedro Hispano Hospital, 4464-513 Matosinhos, Portugal; cgavina@med.up.pt; 2 Department of Community Medicine, Information and Decision in Health, Faculty of Medicine, University of Porto, 4050-313 Porto, Portugal; up202103237@edu.med.up.pt (D.S.C.); dmdias@med.up.pt (D.M.D.); tiagogomes@med.up.pt (T.T.-G.); 3 Family Health Unit Ao Encontro da Saúde, ACeS Grande Porto I-Santo Tirso/Trofa, 4745-559 Trofa, Portugal; 4 Medical Department, AstraZeneca, 2730-097 Barbarena, Portugal; filipa.bernardo@astrazeneca.com (F.B.); hugo.martinho@astrazeneca.com (H.M.); joao.couceiro@astrazeneca.com (J.C.); 5 Nephrology Department, Pedro Hispano Hospital, 4464-513 Matosinhos, Portugal; csaraujo@med.up.pt; 6 UnIC@RISE, Department of Surgery and Physiology, Faculty of Medicine, University of Porto, 4200-319 Porto, Portugal; 7 TOXRUN–Toxicology Research Unit, University Institute of Health Sciences, Advanced Polytechnic and University Cooperative (CESPU), CRL, 4585-116 Gandra, Portugal; 8 Department of Public Health and Forensic Sciences and Medical Education, Faculty of Medicine, University of Porto, 4200-319 Porto, Portugal; 9 UCIBIO-REQUIMTE, Laboratory of Toxicology, Department of Biological Sciences, Faculty of Pharmacy, University of Porto, 4050-313 Porto, Portugal

**Keywords:** early mortality, diabetes, real-world data, comorbidity, MACE

## Abstract

Introduction: Type 2 diabetes mellitus (T2D) increases the risk of heart failure (HF) and chronic kidney disease (CKD). Nonetheless, evidence of cardiovascular (CV) prognosis is relatively scarce in young T2D patients. Purpose: To estimate the risk of all-cause death, CV death, and non-fatal major CV events (MACEs) in T2D patients younger than 65 years old. Methods: We designed a retrospective cohort study using incident cases of either T2D, HF, or CKD in the population aged 40–65 years, from 1st January 2000 to 31st December 2019. Each individual was followed for up to one year. The primary analysis consisted of survival analysis with Cox proportional hazards to compare one-year risk of all-cause death, CV death, and MACEs between T2D without HF or CKD (T2D), T2D with HF (T2D-HF), and T2D with CKD (T2D-CKD) groups. Results: A total of 14,986 incident adult diabetic patients from the last two decades in our institution were included with an average age at cohort inclusion of 55–58 years old. Glycemic control was similar among groups. The adjusted hazard ratio (HR) of one-year all-cause death was 2.77 (95% CI: 2.26–3.40) for T2D-HF and 3.09 (2.77–3.45) for T2D-CKD compared with the baseline T2D risk. The highest event rate (T2D-CKD) was 0.15 per person-year. The adjusted HR of one-year CV death was 2.75 (95% CI: 2.19–3.46) for T2D-CKD and 2.59 (1.72–3.91) for T2D-HF. The non-fatal MACE risk was significantly increased in T2D-HF or T2D-CKD compared with T2D (2.82 (CI95%: 2.34–3.41) for T2D-CKD vs. 1.90 (CI95%: 1.66–2.17) for T2D-CKD) with a 32% event rate in non-fatal MACEs. Conclusions: Coexistence of HF or CKD is associated with increased premature mortality as well as non-fatal CV events in T2D patients under 65 years old.

## 1. Introduction

Type 2 diabetes mellitus (T2D) has become a major global public health concern with a significant clinical and socio-economic burden [1,2,3]. In Portugal, diabetes affects 13.6% of individuals older than 20 years and is more prevalent in men. In the past decade, T2D’s prevalence has increased by 1.9%, highly driven by the aging of the population [4]. Diabetes is a leading cause of death and was responsible for 4200 Years of Potential Life Lost (YLL) in the population below 70 years old, representing an estimated direct cost between 0.6 and 0.8% of the Portuguese gross domestic product (GDP) and 7 to 8% of the health expenditure, in 2018 [4]. T2D induces macrovascular and/or microvascular pathological changes that increase the risk of heart failure (HF) and chronic kidney disease (CKD) and represents one of the *primum movens* of the cardiorenal continuum [5]. The interconnection between these three entities is well documented, sharing pathophysiological mechanisms that potentiate each other [6,7].

Early onset T2D has a significant clinical impact. Due to a longer lifetime exposure to hyperglycemia, young T2D patients are prone to vascular complications [8] and have a much higher risk of developing cardiovascular (CV) disease [9], especially patients less than 65 years old [10]. Previous studies on middle-aged people (a mean age of 49 to 69 years) with diabetes demonstrated that between 2 and 27 in 1000 patients die from CV diseases yearly [11]. This significantly affects life expectancy, with an estimated YLL of 1.7 years/average person [12].

Understanding the connection between T2D and HF or CKD regarding CV outcomes grants an appropriate intervention at an earlier stage of the disease to improve the long-term prognosis for people with diabetes [13]. Despite being a highly prevalent condition, evidence of CV disease prognosis is relatively scarce regarding young T2D patients. In this study, we aimed to estimate the risk of all-cause death, CV death, and non-fatal major CV events (MACEs) in T2D patients younger than 65 years old, either: (i) without HF or CKD; (ii) with HF (T2D-HF); or (iii) with CKD (T2D-CKD), in a real-world clinical setting.

## 2. Materials and Methods

### 2.1. Study Design and Participant Selection

**Figure 1:**
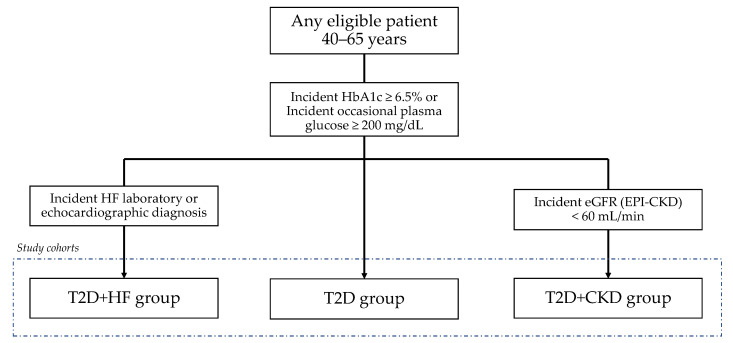
Flowchart of the study cohort design allowing for the simultaneous inclusion of all eligible patients aged 40–65 years. HF, heart failure; CKD-EPI, chronic kidney disease epidemiology collaboration; T2D, diabetes mellitus type 2; HbA1c, hemoglobin A1c; eGFR, estimated glomerular filtration rate.

This is a non-interventional longitudinal study performed in the Health Local Unit of Matosinhos (Unidade Local de Saúde de Matosinhos (ULSM)), a regional health system in the district of Matosinhos in the north of Portugal, encompassing 14 Primary Care Health Units (PCHUs) assisted by the same Secondary and Tertiary Care Health Unit (STCHU) at Pedro Hispano Hospital. Incident cases of either T2D, HF, or CKD in the population aged 40–65 years, from 1st January 2000 to 31st December 2019, as defined by the below-described criteria, were deemed eligible for cohort inclusion. T2D incident cases were eligible for direct cohort inclusion in the T2D group whilst cases of HF and CKD onset with prior or consecutive T2D development were deemed eligible for inclusion in the T2D-HF and T2D-CKD groups, respectively. Incident cases were censored after completing one year of follow-up or upon death (Figure 1). The exclusion criteria were: less than one year of clinical data recorded prior to disease onset; and no primary care appointments in the last three years prior to disease onset. Data access for analysis was granted after approval by the Ethical Committee and Data Protection Officer of the Health Unit (translated from the Comissão de Ética para a Saúde da Unidade Local de Saúde de Matosinhos) (approval code Nº34/CE/JAS of 23-04-2020 (original) and Nº64/CE/JAS of 10-07-2020 (addenda)). De-identified data were extracted from electronic heath records according to the HIPAA Safe Harbor Standard. Data regarding age, gender, and comorbidities were classified by the International Classification of Diseases (ICD)-9 and 10 codes, and medications were registered according to the Anatomical Therapeutic Chemical Classification System.

### 2.2. Type 2 Diabetes Mellitus Inclusion Criteria

A definite diagnosis of T2D was defined as any registered measurement of HbA1c ≥ 6.5% or a measurement of occasional plasma glucose ≥ 200 mg/dL. A fasting blood sugar of at least 126 mg/dL was not included as a diagnostic criterion since it was not possible to ensure that all patients attending the 14 PCHUs and the STCHU enrolled in this study were fasting at the time of blood sampling.

### 2.3. Heart Failure Inclusion Criteria

HF diagnosis was based on laboratory and echocardiographic parameters adapted from HF diagnostic criteria implemented in two recent HF trials covering HFpEF and HFrEF: PARAGON-HF [14] and DAPA-HF [15]. More specifically, patients needed to meet either the HF with a reduced ejection fraction (EF) or HF with a non-reduced EF criteria. HF with reduced EF needed an echocardiographic EF ≤ 40% and either NT-proBNP ≥ 200 pg/mL (≥600 pg/mL in the presence of atrial fibrillation (AF)) or BNP ≥ 100 pg/mL (≥125 pg/mL in the presence of AF). HF with non-reduced EF was defined as EF > 40% in the presence of one of the following structural cardiac abnormalities: left atrial (LA) volume index > 30 mL/m^2^; LA volume > 50 mL; LA diameter > 38 mm; interventricular septum (IVS) > 11 mm; posterior wall (PW) > 11 mm; and left ventricular mass index (LVMi) > 115 g/m^2^ for men or > 95 g/m^2^ for women.

### 2.4. Chronic Kidney Disease Inclusion Criteria

CKD was defined as having at least one measurement of eGFR < 60 mL/min. eGFR was calculated using the EPI-CKD formula (Levey et al. 2009) and used to stage CKD. To allow for incident cases only, at least one prior documented measurement of eGFR > 60 mL/min was required.

### 2.5. Additional Definitions

Myocardial infarction, hypertension, AF, stroke, peripheral artery disease, and microvascular disease were defined by the presence of at least one ICPC-2, ICD-9, or ICD-10 code.

### 2.6. Outcome Measurements

Evaluated outcomes included all-cause death, cardiovascular death, and major adverse cardiac events (a composite outcome of myocardial infarction, stroke, peripheral artery disease, and end-stage CKD).

### 2.7. Data Collection and Statistical Analysis

Data analysis took place between June and September 2020. eGFR and indexed echocardiographic parameters were computed directly from available lab and measurement data. Continuous variables are reported using the mean and standard deviation (SD) or the median and interquartile range (IQR), while categorical variables are reported using absolute and relative frequencies, as appropriate. The primary analysis consisted of survival analysis with Cox proportional hazard models using age as a splitter variable and adjusting for sex–age and hypertension–comorbidity interactions. Models were clustered by patient id to permit cohort simultaneity. Statistical analysis was performed using Apache Spark Framework version 2.45 and R version 4.03. *p* values < 0.05 were considered significant.

### 2.8. Ethical Approval

Ethical and data privacy clearance were granted by the Ethics Committee and Data Protection Officer of the institution.

## 3. Results

A total of 14,986 incident adult diabetic patients from the last two decades in our institution were included, with an average age on T2D diagnosis of 55.69 ± 6.58 years. Of these, 1101 simultaneously met the defined criteria for HF and 3114 met the criteria for CKD. The average age at CKD and HF onset was approximately 3 years older than in pure T2D (please see Table 1 for the baseline characteristics of the cohorts).

### 3.1. T2D Cohort

These individuals presented a mean age of 55.69 years old at onset. The most common comorbidity was hypertension (31.92%). Microvascular disease reached 2% at onset. Regarding glucose control, patients had a median HbA1c value of 6.9% at diagnosis. At baseline, BNP and pro-BNP levels were lower than in the TD2-HF and TD2-CKD cohorts. The most-administered antidiabetic drug was metformin (24.46%) followed by sulfonylureas (8.65%). Less than 2% of patients were insulin-treated (1.66%). Statins, ACE inhibitors, and beta-blockers were the most-prescribed drug classes.

### 3.2. T2D and HF Cohort

At the cohort index date, the average age in this group was 58.3 years old with a high prevalence of comorbidities, namely hypertension (68.21%), stable (23.61%) and unstable (8.08%) angina, and atrial fibrillation (17.9%). Microvascular disease was present in over one-fifth of the patients (20.6%), and a history of myocardial infarction and stroke was found in 28.52% and 18.80% of the patients, respectively.

HF with a non-reduced left ventricular ejection fraction (EF) was more prevalent (20.89%) than HF with a mildly reduced EF (6.54%) and HF with a reduced EF (9.17%), with an overall median EF of 55% (IQR: 34.75–75.25). The median BNP and NT-pro-BNP were 273 and 3732 pg/mL, respectively. Glucose control was similar to that of the overall T2D group (7%).

The most common oral diabetes treatment at baseline was metformin (40.33% of patients), followed by sulfonylureas (24.16%) and DPP-4 inhibitors (22.25%). Nearly one-quarter of the patients were prescribed an insulin regimen (19.80%). More than half of these patients were prescribed a statin. ACE inhibitors, beta blockers, diuretics, and low-dose aspirin were each prescribed to over 40% of the cohort.

### 3.3. T2D and CKD Cohort

This cohort had a mean age of 58.36 years old and a male gender predominance (54.3%). Hypertension was by far the most common comorbidity (59.57%), followed by a history of stroke (9.60%), stable angina (7.29%), and a history of myocardial infarction (6.42%). Microvascular disease neared one-tenth of the cohort at the follow-up starting point (9.4%). Glycemic control at baseline showed a median HbA1c value of 7.1%, similar to the other cohorts.

The overall median eGFR was 53.4 mL/min, with a median serum creatinine value of 1.4 mg/dL. Stage 3 CKD was the most prevalent stage (G3a and G3b accounted for 56% and 14% of the overall renal function of the cohort, respectively). The more advanced stages (G4 and G5) were less common (6% and 1%, respectively).

Metformin was the most frequently prescribed oral glucose-lowering drug (48.01%), followed by sulfonylureas and DPP-4 inhibitors (26.53 and 22.67%, respectively). Patients on insulin therapy accounted for a total of 12.81% of the cohort. Statins were prescribed to nearly half of the patients (48.94%), followed by ACE inhibitors (40.2%), low-dose aspirin (25.37%), beta blockers (25.37%), and diuretics (18.08%).

### 3.4. Mortality Risk

**Figure 2:**
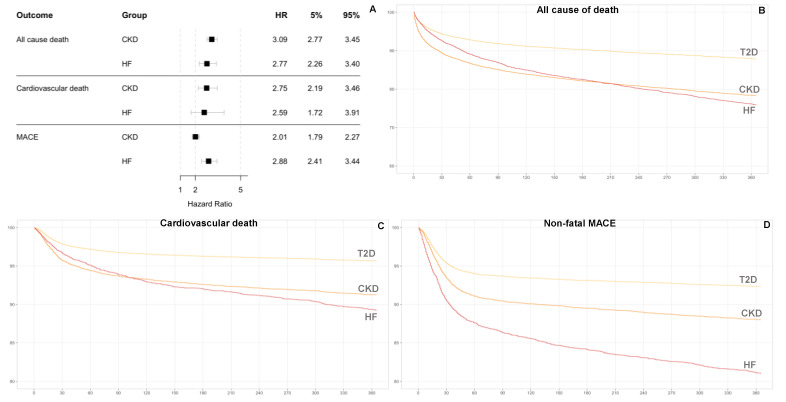
Adjusted relative hazard ratios (HRs) for chronic kidney disease (CKD) and heart failure (HF) using diabetes mellitus type 2 (T2D) (**A**), Kaplan–Meier survival curves for risk comparison regarding all-cause deaths (**B**), cardiovascular deaths (**C**), and major adverse cardiac events (MACEs) (**D**) at one year of follow-up as outcomes.

The cohort with the highest all-cause mortality event rate at one-year follow-up was the T2D plus CKD cohort (0.15 per person-year), followed by the T2D with HF cohort (0.14 per person-year). The T2D group had the lowest all-cause mortality rate: 0.05 per person-year. The adjusted hazard ratio (HR) of one-year all-cause death was 2.77 (95% CI: 2.26–3.40) for T2D-HF and 3.09 (2.77–3.45) for T2D-CKD compared with T2D (Table 2, Figure 2A,B).

Regarding death by a cardiovascular event, the one-year event rate was 4% in the T2D with HF group, 3% in the T2D with CKD group, and 1% in patients with only T2D. The adjusted HR of one-year cardiovascular death was 2.75 (95% CI: 2.19–3.46) for T2D-CKD patients and 2.59 (1.72–3.91) for T2D-HF patients compared with T2D patients without those conditions (Table 2, Figure 2A,C).

### 3.5. Non-Fatal MACEs

The non-fatal MACE risk was significantly increased in T2D-HF and T2D-CKD patients compared with T2D patients and was higher in patients with T2D-HF than in patients with T2D-CKD (2.82 (CI95%: 2.34–3.41) for T2D-HF vs. 1.90 (CI95%: 1.66–2.17) for T2D-CKD) (Table 2, Figure 2A,D). Regarding non-fatal MACEs, the event rate was 32% in the T2D-HF cohort, 13% in the T2D-CKD cohort, and only 6% in the T2D-only cohort.

## 4. Discussion

T2D is a risk factor for both CKD and HF development and their mutual tripartite relationship is increasingly being recognized [16]. Their onset leads to a synergistic effect that increases patient morbidity and mortality [16]. The CKD and HF subgroups both had a higher prevalence of cardiovascular comorbidities and use of cardiovascular medications. Our study demonstrated that the HF and CKD comorbidities are associated with a premature mortality rate at one year (both all-cause and cardiovascular) that is higher than the T2D baseline expected risk. Concerning MACEs, we also observed a substantially higher event rate in T2D-HF compared with T2D-CKD. Our findings are in line with previous reports [13], providing further evidence and quantifying the interaction between conditions regarding short-term mortality risk in a relatively young patient cohort. The pathophysiology may be related to cardiorenal interactions, of which cardiorenal syndrome, a clinical entity in which the primary event is heart injury followed by progressive kidney damage, is an example [17,18].

It is important to highlight the relatively low use of oral glucose-lowering drugs with demonstrated cardiovascular and renal benefits in patients with T2D and CKD or HF that have been shown to prevent or delay the progression of HF and CKD [15,19,20]. Nonetheless, most of the person-years at risk were included before the availability of SGLT-2 inhibitors and GLP-1 receptor agonists. Specifically, developments in the pharmacological class of SGLT2 inhibitors over the last few years have rendered them promising in the treatment of DM [21], in the treatment of HF (with both a reduced [19] and a preserved [22,23] ejection fraction), and in the improvement of CKD outcomes [20,24]. Nevertheless, we must stress that our study design is intended to accurately determine baseline risks and not to study drug efficiency.

At least two limitations need to be highlighted. Firstly, the conducted analysis was based on the data available in electronic health records with their unavoidable potential for data quality problems, and the study was performed at a single center serving an urban population, which may impair as previously demonstrated [16] the direct translation of prevalence estimations to other populations. Moreover, our CKD definition did not include multiple eGRF measurements below 60 mL/min/1.73 m^2^ for a duration greater than 3 months. Nevertheless, ongoing studies from our research group have obtained comparable estimates using two eGFR measurements versus a single eGRF estimation (differences of 1.3%) (a preprint is available at [25]).

Nonetheless, our results have a relevant clinical impact. Specifically, the study design was focused on obtaining an accurate patient cohort for an adequate risk comparison by including only incident cases rather than focusing on an adequate prevalence estimation. Additionally, we included patients based on contemporary disease definitions rather than relying solely on health professionals’ coding. Particularly, natriuretic peptides and echocardiographic data on individuals were included for HF diagnosis, and CKD stages were derived from the estimation of GFR using the CKD-EPI equation [26]. Our study includes two decades’ worth of real-world patient data (over 15,000 person-years at risk) on short-term outcomes under different therapeutic approaches and compares groups with similar glycemic control values. This strengthens the findings and probably closely identifies the true magnitude of the associations found, which was previously unknown for Portugal. Another advantage of our study is that it was driven by a health unit that integrates both primary, secondary, and tertiary health care units; thus, our cohort more closely represents the general diabetic population than cohorts from only one of those settings separately.

In conclusion, our results suggest that HF and CKD are important outcome predictors in this T2D patient subset, with over two-fold increases in all-cause and cardiovascular mortality. Therefore, we advocate for the raising of awareness among clinicians about screening for both HF and CKD in young patients with T2D.

## Figures and Tables

**Table 1:** Baseline characteristics of the cohorts at the onset of diabetes mellitus type 2, heart failure, and chronic kidney disease.

	T2D (*n* = 14,986)	T2D + HF (*n* = 1101)	T2D + CKD (*n* = 3114)
Demographics			
Age (years)—mean (SD)	55.69 (6.58)	58.32 (5.83)	58.36 (5.96)
Sex (males)—*n* (%)	8486 (56.63)	667 (60.58)	1692 (54.34)
General comorbidities—*n* (%)			
Hypertension	4783 (31.92)	51 (68.21)	1855 (59.57)
Cardiovascular disease	1095 (7.31)	674 (61.22)	668 (21.45)
Cardiorenal disease	284 (1.9)	775 (70.39)	264 (8.48)
Myocardial infarction	370 (2.47)	314 (28.52)	200 (6.42)
Unstable angina	125 (0.83)	89 (8.08)	89 (2.86)
Stable angina	325 (2.17)	260 (23.61)	227 (7.29)
Atrial fibrillation	159 (1.06)	197 (17.89)	120 (3.85)
Stroke	475 (3.17)	207 (18.8)	299 (9.6)
Peripheral artery disease	41 (0.27)	53 (4.81)	44 (1.41)
Microvascular disease	301 (2.01)	227 (20.62)	294 (9.44)
Clinical assessment—median (IQR)			
Systolic blood pressure (mmHg)	138 (20)	137 (26)	138 (22)
Diastolic blood pressure (mmHg)	85 (11)	80 (15)	82 (14)
Body mass index (kg/m^2^)	29.98 (6.55)	29.75 (7.46)	30.07 (6.69)
Waist circumference (cm)	102 (15)	104 (17.75)	104 (17)
Laboratory assessment—median (IQR)			
Glycated hemoglobin (%)	6.9 (1.3)	7 (1.9)	7.1 (1.9)
LDL cholesterol (mg/dL)	121.73 (52)	109 (53)	113.8 (54)
HDL cholesterol (mg/dL)	43 (16)	38 (16.15)	41 (16.2)
Total cholesterol (mg/dL)	201 (60)	176 (61.1)	188 (64.32)
Triglycerides (mg/dL)	145 (103)	126 (93)	148 (105)
BNP (pg/mL)	123.1 (298.6)	273.1 (560.7)	124.2 (428.2)
NT-proBNP (pg/mL)	377.9 (1589.85)	3732 (5193.8)	6036.15 (2304.15)
Serum creatinine (mg/mL)	0.8 (0.3)	0.9 (0.6)	1.4 (0.6)
Albumin to Creatinine Ratio (mg/g)	12.2 (22.2)	32 (216.91)	17 (65.5)
eGFR (mL/min, MDRD)	91.5 (31.02)	79.25 (54.66)	53.41 (18.26)
Baseline medication, diabetes—*n* (%)			
Oral antidiabetics	4128 (27.55)	504 (45.78)	1608 (51.64)
Insulins	249 (1.66)	218 (19.8)	399 (12.81)
Metformin	3636 (24.26)	444 (40.33)	1495 (48.01)
SGLT-2 inhibitors	20 (0.13)	18 (1.63)	67 (2.15)
DPP-4 inhibitors	745 (4.97)	245 (22.25)	706 (22.67)
Sulfonylurea	1297 (8.65)	266 (24.16)	826 (26.53)
GLP-1 receptor agonists	24 (0.16)	11 (1)	45 (1.45)
Meglitinides	236 (1.57)	60 (5.45)	214 (6.87)
Glitazones	62 (0.41)	24 (2.18)	98 (3.15)
Acarbose	265 (1.77)	62 (5.63)	204 (6.55)

T2D, diabetes mellitus type 2; HF, heart failure; CKD, chronic kidney disease; LDL, low-density lipoprotein; HDL, high-density lipoprotein; MDRD, Modification of Diet in Renal Disease; SGLT2i, sodium-glucose co-transporter-2 inhibitors; GLP-1 RA, glucagon-like peptide-1 receptor agonists; DPP-4i, dipeptidyl peptidase 4 inhibitors; GLP-1 RAs, glucagon-like peptide-1 receptor agonists; BNP, B-type natriuretic peptide; eGFR, estimated glomerular filtration rate; IRQ, interquartile range.

**Table 2:** Weighted means of hazard ratios (HRs) for all-cause-death, cardiovascular death, and major adverse cardiovascular events (MACEs).

		Patients (*n*)	Person-Years Cohort Total	Event Rate per Person-Year	Follow-Up (Days) Mean (SD)	Time to Event Mean (SD)	HR (95% CI)
All Cause Death	T2D	14,123	12,572.37	0.05	324.92 (102.72)	91.91 (105.15)	-
	T2D + CKD	3002	2502.96	0.15	304.44 (121.20)	77.93 (96.49)	3.09 (2.77–3.45)
	T2D + HF	1003	842.36	0.14	306.54 (115.05)	126.26 (113.86)	2.77 (2.26–3.40)
Cardiovascular death	T2D	14,179	12,632.98	0.01	325.20 (101.94)	62.24 (81.09)	-
	T2D + CKD	3035	2531.41	0.03	304.44 (119.86)	69.71 (84.56)	2.75 (2.19–3.46)
	T2D + HF	1012	852.26	0.04	307.39 (114.15)	111.16 (112.87)	2.59 (1.72–3.91)
MACEs	T2D	14,179	12,214.51	0.06	314.43 (114.42)	49.89 (73.27)	-
	T2D + CKD	3035	2371.61	0.13	285.22 (134.50)	56.44 (73.07)	2.01 (1.79–2.27)
	T2D + HF	1012	715.87	0.32	258.19 (147.54)	66.06 (90.34)	2.88 (2.41–3.44)

HR, hazard ratio; T2D, diabetes mellitus type 2; CKD, chronic kidney disease; HF, heart failure; MACEs, major adverse cardiac events.
